# Universal Cardiovascular Disease Risk Assessment in Pregnancy: Call to Action *JACC: Advances* Expert Panel

**DOI:** 10.1016/j.jacadv.2024.101055

**Published:** 2024-06-23

**Authors:** Afshan B. Hameed, Maryam Tarsa, Cornelia R. Graves, Anna Grodzinsky, Heike Thiel De Bocanegra, Diana S. Wolfe

**Affiliations:** a Maternal-Fetal Medicine, Obstetrics & Gynecology, Cardiology, University of California, Irvine, California, USA; b Division of Maternal-Fetal Medicine, University of California, San Diego, California, USA; c Division of Maternal-Fetal Medicine, University of Tennessee, Nashville, Tennessee, USA; d Cardiology, Saint Luke’s Mid America Heart Institute, University of Missouri-Kansas City, Kansas City, Missouri, USA; e Albert Einstein College of Medicine and Montefiore Medical Center, Bronx, New York, USA

**Keywords:** cardio-obstetrics, cardiovascular disease, high-risk pregnancy, pregnancy, universal CVD risk assessment

## Abstract

The United States has the highest maternal mortality rate among developed countries, with cardiovascular disease (CVD) being one of the leading causes of maternal deaths. Diagnosing CVD during pregnancy may be challenging as symptoms of normal pregnancy overlap with those of CVD. Delays in recognition and response to the diagnosis of CVD is a missed opportunity for timely intervention to improve maternal outcomes. Implementing universal CVD risk assessment for all pregnant and postpartum patients across clinical care settings presents a pivotal opportunity to address this issue. Integrating a validated risk assessment tool into routine obstetric care, clinicians, including obstetricians, primary care, and emergency healthcare providers, can enhance awareness of cardiovascular risk and facilitate early CVD diagnosis. Consensus among stakeholders underscores the importance of screening and education on cardiovascular health strategies for pregnant and postpartum patients to reduce CVD-related maternal mortality. This comprehensive approach offers a pathway to identify at-risk individuals and intervene promptly, potentially saving lives and advancing maternal healthcare equity.

## Highlights

• Pregnancy symptoms mimic those of cardiovascular disease and therefore diagnosis may be delayed or missed contributing to increase in maternal morbidity and/or mortality.
• Universal cardiovascular risk assessment may help identify previously undiagnosed cardiac disease and new onset cardiomyopathy during pregnancy and/or postpartum period.
• Cardiovascular risk assessment in pregnancy and/or postpartum period must be a priority in education, clinical care, research, and policy development.

## The problem

The United States has the highest maternal mortality rate among the developed countries. The number continues to rise, with cardiovascular disease (CVD) contributing to one-quarter of the maternal deaths. Diagnosis of CVD is challenging during pregnancy due to the overlap of symptoms of normal pregnancy. Therefore, delays in recognition and response remain the primary drivers of adverse maternal outcomes related to CVD.

## A solution

Consensus obtained through development of CVD toolkits and patient safety bundles indicate that the key step to lowering cardiovascular-related maternal mortality is the implementation of screening and clinician and patient education on cardiovascular health strategies in all care settings for pregnant and postpartum patients. Integration of a cardiovascular risk assessment tool to routine pregnancy/postpartum care may identify patients at risk of complications allowing for timely interventions. These patients may not otherwise be alerted of their increased CVD risk or get diagnosed with underlying CVD during pregnancy, delivery, hospitalization, or during the postpartum period (Central Illustration).

**Central Illustration:**
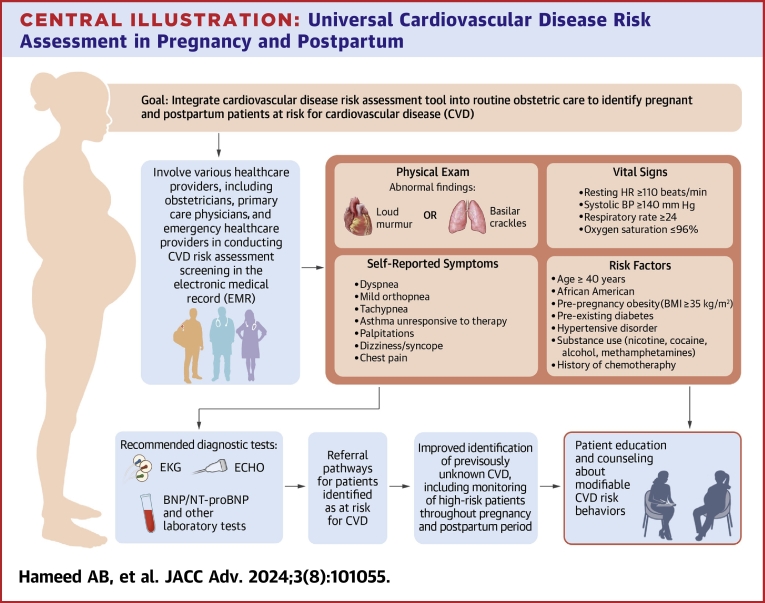
**Universal Cardiovascular Disease Risk Assessment in Pregnancy and Postpartum** The process of integrating cardiovascular disease (CVD) risk assessment begins by involving healthcare providers during the pregnancy and postpartum period to conduct and complete the risk assessment. This assessment captures patient symptom data, vital signs, physical exam data, and CVD risk factors. Based on the risk assessment output, patients could be identified as 'at risk' for CVD, and the algorithm recommends follow-up diagnostic tests and referral pathways, as well as continued patient education and counseling. EKG = electrocardiogram; ECHO = echocardiogram; BNP = B-type natriuretic peptide; NT-proBNP = N-terminal pro-brain natriuretic peptide.

## Background

The United States has the highest maternal mortality rate among developed countries despite numerous initiatives and efforts to improve maternal health.^1^^,^^2^ A recent report by the Centers for Disease Control calls attention to the alarming increase in maternal deaths to 32.9 from 23.8 per 100,000 births in 2020. Significant racial ethnic disparities exist with non-Hispanic black patients (69.9 per 100,000) at 2.6 times risk of maternal death compared to non-Hispanic white patients. Among Hispanic women, the mortality rate increased from 12.6 to 28 per 100,000 live births from 2019 to 2021, with the highest risk observed among women aged 40 and older.^3^ Advancing maternal age increases the risk with mortality rate among mothers ≥40 years of age 6.8 times higher (138.5 deaths per 100,000 live births) than those ≤25 years of age which adds to the existing disparities. Furthermore, Pacific Islanders and Native American and Alaskan Islanders have an increased risk of maternal mortality when compared to non-Hispanic white women.^4^ In fact, since 1999, Native American and Alaskan Islanders have had the largest increase in maternal mortality.^5^ Cardiomyopathy accounted for 14.5% of all deaths in the population presenting as a greater proportionate cause of death than any racial group.^6^ Black people, Indigenous people, and people of color are more likely to encounter perinatal medical and cardiovascular complications, despite comparable social drivers of health impacting their care and outcomes. Implicit bias of providers and perceived racial discrimination from patients impact trust in the health care system, resulting in delayed encounters for screening and reservations about research enrollment. These disparities can be largely attributed to structural and institutional racism which have resulted in social structures and barriers to care rather than biological factors.^7^ Nonetheless, CVD including cardiomyopathy remains accountable for one-quarter of maternal deaths.^8^ One can deduce that racisim is a risk factor for CVD.

It is estimated that 2 out of every 3 pregnancy-related deaths are preventable.^9^ Comparative data of the major causes of maternal mortality indicate that most other causes of maternal deaths have declined over time; however, those due to CVD have increased.^10^ The increased numbers may partly be reflective of the improved Maternal Mortality Reviews (MMR).^11^ Most states in the U.S. now have organized MMR surveillance systems in place with state-wide in-depth analysis on a case-by-case basis.^12^ MMR have proven to be an essential first step in understanding the gaps in healthcare and identification of improvement opportunities.^13^

Stakeholders in the U.S. from the obstetrics, cardiology, anesthesia, nursing, and public health fields contributed to the development of the Cardiovascular Disease Toolkit through California Maternal Quality Care Collaborative (CMQCC) and Cardiac Conditions in Obstetrical Care (CCOC) Patient Safety Bundle by the Alliance for Innovation on Maternal Health with a goal of improving the devastating trends.^14^, ^15^, ^16^ Patient safety bundles provide resources that align with actionable steps in an organized manner that ultimately improve system-wide processes and subsequent patient outcomes. The American Heart Association recommends preconception counseling in patients with CVD risk factors, such as diabetes, hypertension, and obesity to optimize maternal and fetal outcomes.^2^ The consensus statement indicates that screening of pregnant people for cardiac conditions in all care settings is a key step to lower CVD-related maternal mortality.^15^ As a result of these collaborations, a working group discussed strategies for the implementation of universal CVD risk assessment for all pregnant and postpartum people.

## The challenge

Pregnancy is a state of hemodynamic overload that may lead to signs and symptoms that mimic cardiac disease in normal pregnancy.^17^ It may be challenging to differentiate the common complaints of fatigue, shortness of breath, and swelling from those due to CVD. The American College of Obstetrics and Gynecology provides guidance for the evaluation of these symptoms and the urgency for additional evaluation.^18^ Evidence is clear that patients with preexisting congenital and/or acquired CVD benefit through use of risk stratification models that guide management strategies.^19^^,^^20^ Ideally, patients with known CVD should undergo preconception counseling that outlines an individualized treatment plan before pregnancy.^21^ Evidence-based guidelines exist on the need for referral to the appropriate level of care for cardiac patients.^22^ However, there is a paucity of literature on ways to identify pregnant and/or postpartum patients who may have unrecognized CVD or may be at risk for CVD without a known diagnosis. Reports using the CMQCC cardiovascular risk assessment algorithm and other methodologies using physical examination and electrocardiograms as screening tests for CVD detection in pregnant patients have been published to identify pregnant patients at increased risk of CVD.^23^, ^24^, ^25^, ^26^ Most of the existing literature focuses on postpartum CVD risk assessment in patients with adverse pregnancy outcomes as a surrogate for future CVD.^27^

Early detection and risk stratification of CVD among pregnant and postpartum patients is essential as most patients who died of CVD-related causes had underlying risk factors and had sought healthcare with CVD-related symptoms and/or had vital sign abnormities that were documented but unfortunately attributed to the normal pregnancy-related hemodynamic stress rather than “a red flag” for the presence of underling CVD. Consequently, more than half of the decedents were diagnosed with CVD only at autopsy.^17^

Pregnant patients with CVD may present with a known diagnosis or may have underlying previously unknown CVD that may manifest as onset of symptoms and/or physical examination abnormalities during pregnancy or postpartum period. The value of preconception counseling, early diagnosis, and multidisciplinary care cannot be overemphasized. People with undiagnosed unknown CVD and those with CVD index diagnosis during their pregnancy usually present in a similar manner with symptoms and abnormal vital signs, however, may not be diagnosed in time to receive guideline-recommended medical care.^17^

Often, patients for whom CVD is diagnosed for the first time in pregnancy are pediatric patients who never transitioned to adult care and may not have received well-woman examination and counseling. To complicate it further, pregnancies may be unplanned without the benefit of contraception or preconception counseling leading to delays in seeking prenatal care.^19^ The other cohort are those with de novo onset of cardiomyopathy, that is, peripartum cardiomyopathy (PPCM), which typically presents with heart failure with reduced ejection fraction toward the end of pregnancy or in the first 5 months postpartum.^28^ Patients who are diagnosed later in pregnancy or during the postpartum period with PPCM often enter pregnancy in their normal state of health with or without CVD risk factors and no evidence of underlying structural cardiac defect.^29^

Knowledge of the underlying cardiac condition is the key to optimize maternal outcomes in a multidisciplinary manner. The role of a “Pregnancy Heart Team” also known as the Cardio-Obstetric Team is integral to the care of this high-risk group of patients.^18^^,^^30^ The key team members include Maternal Fetal Medicine/Obstetrics, Cardiology (Heart Failure, Congenital Heart disease, interventional, electrophysiology specialists), Anesthesia (Obstetrics and cardiothoracic), Neonatology, Nursing Leadership, Patient Safety, Blood Bank, Perfusion Specialists, and for certain cases, Cardiothoracic Surgery.^31^ Typically, the case is presented (history of present illness, physical examination, obstetric complications, and comorbidities) by the obstetrics team, cardiac diagnostic studies shared by cardiology, and the anesthesiologist outlines a safe regional anesthesia plan. Labor and delivery nursing coordinates 1:1 care of the cardiac patient and arranges for additional cardiac expertise on the labor floor as indicated. A checklist for use at these collaborative meetings helps guide members through common management questions, medications safety, and care plan through pregnancy, delivery, and postpartum care that becomes part of the patient’s electronic medical record.

Unfortunately, a large proportion of maternal deaths from CVD are in patients who lack knowledge of their underlying diagnosis of CVD and those with a new diagnosis of CVD not made early enough for treatment and alter outcomes. Delays in recognition and diagnosis are the recurrent theme in mothers who died of CVD.^14^

## A solution

### Early recognition of CVD to allow for timely management is the key element required to lower the rates of maternal morbidity and mortality

Patients with known diagnosis of CVD and those with conventional CVD risk factors without a prior diagnosis of CVD benefit by optimizing health and multidisciplinary pregnancy care at an appropriate level of maternal care.^22^ The role of preconception counseling cannot be overemphasized in this high-risk group of patients. Likewise, earlier recognition of a previously unknown CVD or a timely diagnosis of pregnancy-associated cardiomyopathy is bound to improve maternal and fetal outcomes.^15^ This scenario represents the largest gap in care of pregnant and postpartum patients that drives the CVD contribution to maternal mortality. For pregnant patients with known CVD including those with newly diagnosed PPCM, the modified World Health Organization’s Risk Stratification (ESC 2018 guidelines) provides guidance on percent risk of a Maternal Cardiac Event Rate and delivery location, to help transfer or triage patients to receive the appropriate level of care. Likewise, the AIM CCOC Bundle includes a Change Package that outlines under the “R” Response a change concept: Coordinate transitions of care, especially in the “fourth” trimester, including the discharge from the birthing facility to home and transition from postpartum care to ongoing primary and specialty care along with the resources and tools for institutions to utilize and implement in various healthcare settings.^32^

**Figure 1:**
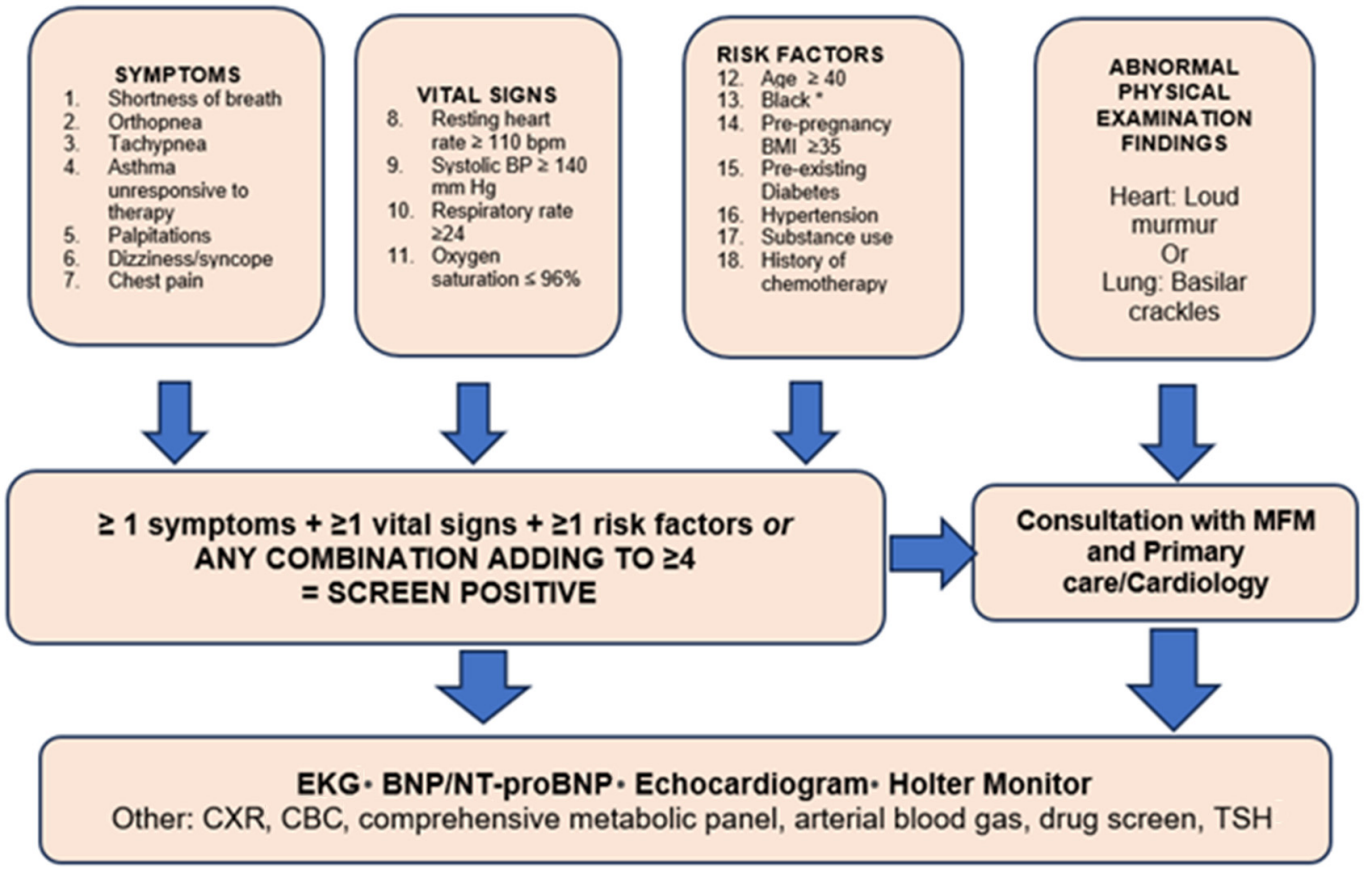
**CVD Risk Assessment Algorithm** The algorithm is organized in the form of 4 buckets and identify patients who present with a cluster of variables as being at risk for CVD. One variable from each of the first 3 buckets or 4 variables from any of the first 3 buckets deems the patient at increased risk for CVD. The fourth bucket, that is, abnormal physical examination findings in itself identifies the patient at increased risk for CVD that requires additional cardiac work up. ∗This is not related to biological race but secondary to structural racism and inequities in care. BNP = B-type natriuretic peptide/pro-BNP; CBC = complete blood count; CXR = chest radiograph; EKG = electrocardiogram; TSH = thyroid-stimulating Hormone.

We propose universal cardiovascular risk assessment in all pregnant and postpartum patients using the CVD risk assessment algorithm that stratifies pregnant and postpartum patients into the categories of “low risk” and “high risk” for CVD (Figure 1). The tool is based on the clinical presentation and quality improvement opportunities identified in CVD-related maternal deaths and was implemented and evaluated at major hospital systems.^33^^,^^34^ It identifies patients at increased risk of CVD who require further assessment and cardiovascular diagnostic testing. The tool considers the common CVD medical and demographic risk factors, self-reported CVD symptoms, vital signs, and abnormal physical exam findings. The algorithm is organized in the form of 4 buckets shown in Figure 1 that identifies patients who present with a cluster of variables as being at risk for CVD. One of the buckets that constitutes abnormal physical examination findings on heart or lung auscultation is considered high risk for underlying CVD, and regardless of other elements in the remainder of the buckets, further evaluation such as electrocardiogram, echocardiogram, and/or Brain natriuretic peptide (BNP) or N-terminal pro-brain natriuretic peptide (NT-proBNP) is recommended.^33^^,^^34^

The tool is intended for use in all pregnant and post-partum patients at their first encounter or any of the subsequent visits. Risk assessment can be repeated for patients that screen negative at any point during pregnancy or the postpartum period if there is an onset of new symptoms or other indications per the discretion of the health care provider.^35^ Retrospective application of this algorithm to the 64 maternal deaths of the California MMR identified 93% of the decedents at increased risk of CVD requiring further evaluation.^14^ Blumenthal et al demonstrated that prospective application of the CVD risk assessment algorithm to the general obstetrics population identified 5% of patients with cardiac conditions.^24^ Further studies are under way to establish CVD risk assessment in pregnancy and postpartum as a quality measure.^36^ Although cost effectiveness of such a CVD screening model in pregnancy has not been studied, overall healthcare cost has shown to be reduced by early screening and treatment for CVD with improved health outcomes in the general population.^35^ Early detection of CVD through screening of pregnant and postpartum people is recommended by the AIM CCOC bundle that allows for timely interventions. Despite this need, universal CVD risk screening using CMQCC algorithm has only been implemented as part of research in a few institutions. There is a need to develop validated CVD screening tools to bridge this gap in cardio-obstetrics. The outcome analysis of CVD diagnosed through screening in pregnancy/postpartum period and its impact on morbidity and mortality rates is currently premature. However, this is of high priority and included in next steps of analysis within the CVD screening Validation and Qualitative Improvement research projects.

## Step-by step approach

### Step 1: establish universal CVD risk assessment as part of routine obstetric care

**Figure 2:**
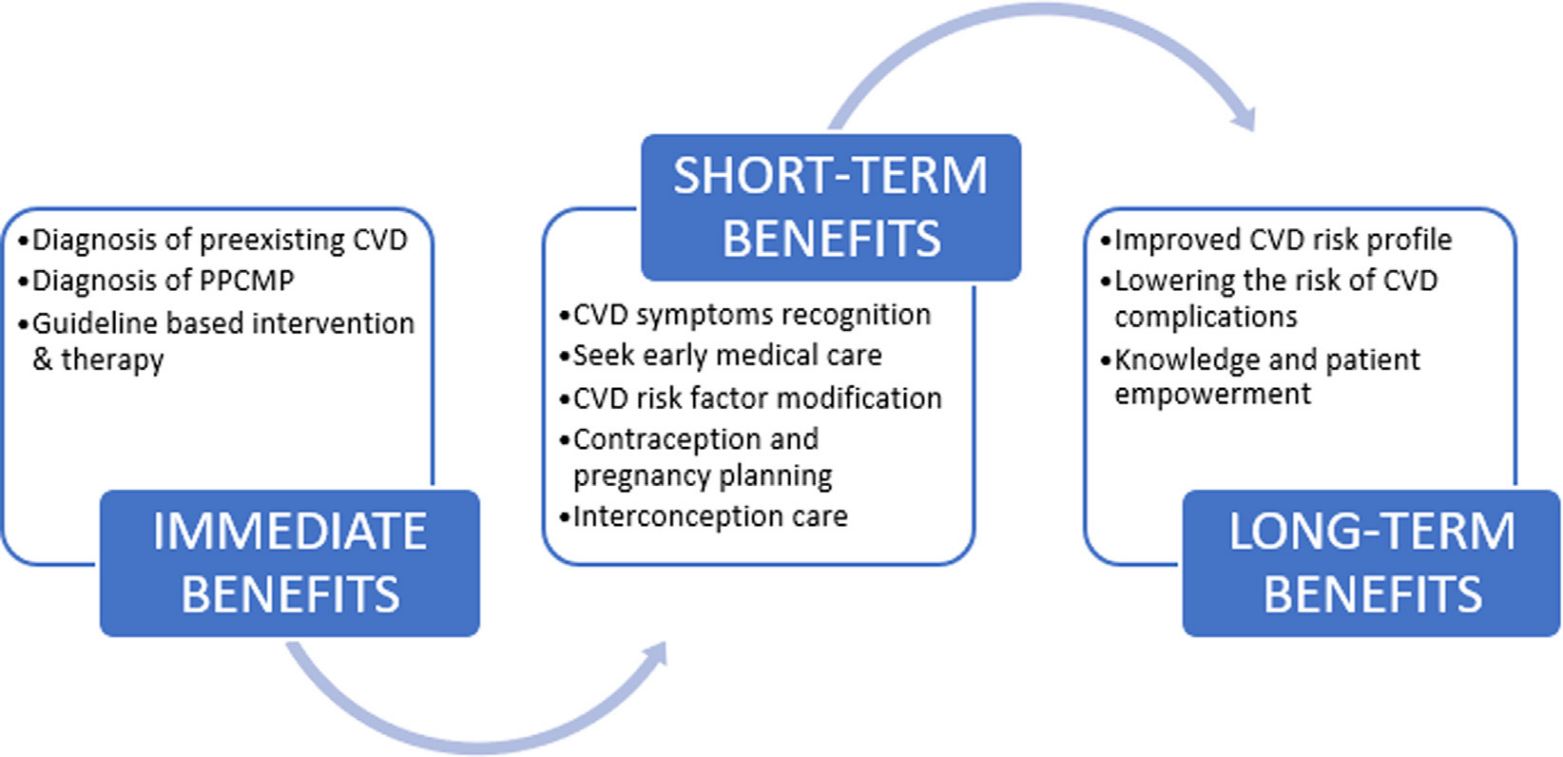
**Benefits of Universal CVD Screening** The immediate, short-term, and long-term benefits for patients identified as high risk for cardiovascular disease (CVD) during pregnancy and postpartum period. PPCM = peripartum cardiomyopathy.

The preferred option is to integrate the CVD algorithm into the electronic medical record (EMR) system at a given hospital (Figure 2). In limited resource clinical settings with a lack or inability to integrate into the EMR, CVD risk assessment can also be performed in a paper checklist format incorporating the elements listed in the risk assessment algorithm. Regardless, successful implementation strategy entails a group of committed individuals from various disciplines (ie, physicians, nurses, staff members, and information technology) who play a crucial role in integrating, implementing, and maintaining the process. Once CVD risk assessment is complete, the patients identified at increased risk of CVD are navigated to a smart set that include the laboratory tests, cardiac diagnostic testing, and referrals that allows the health care provider to place orders for the work up.^28^

### Step 2: how to manage a patient identified at risk for CVD?

Once a patient is identified at increased risk of CVD or CVD risk positive, it is important to share risk assessment results with the patient along with the need for further testing for diagnosis or reassurance. Ideally, care of a patient with CVD begins in the preconception period; however, the diagnosis of CVD may not be known in some patients. The real challenge is the timely identification of previously unknown preexisting CVD or new onset cardiomyopathy in pregnancy/postpartum period. These patients constitute a vast majority of those with catastrophic outcomes. Timely diagnosis is the key as once a patient is diagnosed, ie, true positive CVD screened patients, validated risk prediction tools exist (modified WHO, CARPREG II, ZAHARA) that stratify and guide further management plan. The role of multidisciplinary teams in care for patient with CVD through all stages of care, including pregnancy, labor, delivery, and postpartum cannot be overemphasized. Patient education may include infographics regarding signs and symptoms of CVD that warrant medical attention.^31^ Information regarding cardiac testing as outlined in the algorithm may include an electrocardiogram, echocardiogram, rhythm monitor, laboratory testing including BNP/NT-proBNP, thyroid function testing, and complete blood count. In general, the threshold for the measurement of BNP/NT-proBNP levels should be very low in a pregnant/postpartum patient presenting with cardiac symptoms with or without vital sign abnormalities. A BNP test is inexpensive, readily available with normal values being reassuring with a high negative predictive value for CVD. The patient should be referred to the cardiologist or maternal fetal medicine, a primary care physician, or to the cardio-obstetric clinic (if available) based on the availability of local resources for follow-up. Each health system or unit may modify the process and organize it to best fit their individual resources to make it as efficient as possible. For those persons at highest risk for morbidity and mortality and those who have barriers to healthcare such as lack of insurance or limited health literacy, expeditious evaluation in the hospital is recommended. Community Health Worker programs for patients identified to have positive social determinants of health screening can impact and facilitate navigation of the health system.^37^^,^^38^

### Step 3: the team: reporting and follow-up

The role of the cardio-obstetrics team members is to manage patients who are at increased risk for CVD and to assure that an appropriate work up and referral is completed. This can be done through weekly reporting on all patients with a CVD-positive risk assessment and data tracking to determine appropriate follow up. Administrative support is necessary to assist with timely scheduling to mitigate delays in diagnosis. Availability of telehealth services that were implemented in the post- COVID19 era in many parts of the country can facilitate such practice in hospitals without dedicated cardio-obstetrics teams or areas with limited access to subspecialty care.

## Patient story and perceptions to cardiovascular risk assessment

A crucial part of implementing universal CVD risk assessments is to acknowledge and anticipate patients’ emotional response to increased CVD risk score to help integrate this into counseling and follow-up. While reactions may vary, one of such patient’s narrative illustrates how increased awareness of CVD risk impacts behaviors. Initially unfamiliar with the link between pregnancy and CVD risk, she reported “…*before I got pregnant, I didn’t know that it [cardiovascular disease] was something that pregnant women would have a higher risk of, so now that I do know, I am thinking about it more*”. The absence of prior conversations on this topic within her circle of friends and family contributed to her initial reaction of fear and surprise. The provider's guidance helped her navigate her newfound awareness - “…*she [the doctor] was really reassuring about what we were going to do moving forward and the options, so that made me feel like there was a game plan and ensure that I’m good to go*”. Consequently, she embraced intentional change, altering her diet, increasing physical activity, and even reshaping her family's lifestyle to mitigate potential CVD risks. This transformation extended to meticulous blood pressure monitoring, highlighting her commitment to holistic well-being. For healthcare providers, recognition of these concerns and emotions should become part of counseling and compassionate guidance with the goal of empowering patients to make informed healthcare decisions. To successfully implement universal screening strategies, it is crucial to educate the healthcare providers, ensure clinician buy-in, and integrate patient experience and risk assessment algorithm seamlessly into the standard of care, while keeping clinician’s engagement by incorporating their feedback and their comfort in utilizing these strategies.

## CVD risk assessment as a quality measure

We demonstrated the feasibility of implementing the CVD risk assessment algorithm for pregnant and postpartum patients into EMR at 4 major hospital systems.^32^ The quality measure: *CVD Risk Assessment Measure–Proportion of Pregnant/Postpartum Patients that Receive CVD Risk Assessment with a Standardized Instrument* was included in the 2024 CMS Merit-based Incentive Payment Value Pathways. This will help facilitate the uptake and use in national accountability program.

The quality measure serves for quality improvement initiatives such as Plan-Do-Study-Act (PDSA) cycles. PDSA cycles may aim to improve referrals to higher levels of care and co-management of patients identified to be at risk for CVD, removal of pre-authorizations of follow-up proceedings, and improved patient education. It is important to capture the experience of utilizing the clinical decision support and quality measure in various practice settings, diverse patient demographics, and value of the CVD risk assessment tool for both quality improvement and improved diagnostic excellence purposes, ultimately identifying patients at risk for CVD who may require higher level of care during pregnancy, delivery, and and/or postpartum period.

## Further development of CVD risk assessment tools

We are conducting a validation study that will provide evidence to implement universal CVD risk assessment for all pregnant/postpartum patients at the national level. The study aims to demonstrate the efficiency of the existing CVD screening tool in high-volume clinic settings and to determine the algorithm’s predictive value for the total population and in racial/ethnic subsets to determine the predictive value of each individual variable. The goal is to streamline and simplify the CVD risk assessment tool/algorithm without compromising the predictive value. In the future, additional CVD risk assessment tools may be developed.

Future studies are indicated to evaluate the yield of implementing CVD screening methods as a high number of false-positive test results may put strain on the existing scarce resources. Additionally, it may overwhelm cardio-obstetrics specialists impeding care for the known high-risk CVD patients. Research should also focus on the outcomes, especially the impact on maternal morbidity and mortality due to CVD. Universal CVD screening may prove to be a cost-effective way to prevent CVD-related maternal morbidity and/or mortality.

## Conclusions

Pregnancy is a window of opportunity to address CVD risk, but it is a short timeframe compared to a person’s lifespan. We predict that implementation of universal CVD screening at clinical settings will allow for the identification of preexisting but previously unknown CVD, as well as diagnosis of new onset cardiomyopathy in a timely manner. The use of a CVD risk assessment quality measure and implementation of PDSA cycles allows to identify and address system and clinician barriers. Due to the late pregnancy or postpartum presentation of PPCM, it is imperative that obstetricians work together with the primary care providers, the emergency care providers, and cardiologists to best optimize the best care for pregnant and postpartum patients (Figure 2).^30^

Universal CVD risk assessment would expedite the diagnosis and management of pregnant and postpartum patients who are diagnosed with a cardiac condition because of CVD risk assessment. Timely diagnosis and management of CVD will mitigate their risk of adverse maternal outcomes and ultimately decrease maternal morbidity mortality in the U.S. Additionally, increased patient awareness of the modifiable CVD risk factors has the potential to improve cardiovascular and general health outcomes in birthing people with chronic medical conditions throughout their lifespan.

This is a call to action for the Society for Maternal Fetal Medicine, American College of Obstetrics and Gynecology, the American Heart Association, the American College of Cardiology, and all key stakeholders in the women’s healthcare space to make cardiovascular risk assessment in pregnancy and postpartum period a priority in education, research, and policy development.

## Funding support and author disclosures

This initiative was funded by the 10.13039/100000936Gordon and Betty Moore Foundation, Diagnostic Excellence Initiative, award GBMF9055.01, and from the 10.13039/100000071National Institute of Child Health and Human Development Study #5R21HD101783. The authors have reported that they have no relationships relevant to the contents of this paper to disclose.
